# Activin A Induces Langerhans Cell Differentiation In Vitro and in Human Skin Explants

**DOI:** 10.1371/journal.pone.0003271

**Published:** 2008-09-24

**Authors:** Tiziana Musso, Sara Scutera, William Vermi, Roberta Daniele, Michele Fornaro, Carlotta Castagnoli, Daniela Alotto, Maria Ravanini, Irene Cambieri, Laura Salogni, Angela Rita Elia, Mirella Giovarelli, Fabio Facchetti, Giampiero Girolomoni, Silvano Sozzani

**Affiliations:** 1 Department of Public Health and Microbiology, University of Torino, Turin, Italy; 2 Department of Pathology, University of Brescia, Brescia, Italy; 3 Section of General Pathology and Immunology, Department of Biomedical Sciences and Biotecnology, University of Brescia, Brescia, Italy; 4 Deparment of Dermatology, University of Verona, Verona, Italy; 5 Department of Clinical and Biological Sciences, San Luigi Hospital, University of Torino, Orbassano, Italy; 6 Department of Plastic Surgery and Burn Unit Skin Bank, CTO Hospital, Turin, Italy; 7 Medicine and Experimental Oncology, and Clinical and Biological Sciences, University of Torino, Center for Experimental Research and Medical Studies (CERMS), S. Giovanni Battista Hospital, Turin, Italy; The University of Queensland, Australia

## Abstract

Langerhans cells (LC) represent a well characterized subset of dendritic cells located in the epidermis of skin and mucosae. In vivo, they originate from resident and blood-borne precursors in the presence of keratinocyte-derived TGFβ. Ιn vitro, LC can be generated from monocytes in the presence of GM-CSF, IL-4 and TGFβ. However, the signals that induce LC during an inflammatory reaction are not fully investigated. Here we report that Activin A, a TGFβ family member induced by pro-inflammatory cytokines and involved in skin morphogenesis and wound healing, induces the differentiation of human monocytes into LC in the absence of TGFβ. Activin A-induced LC are Langerin^+^, Birbeck granules^+^, E-cadherin^+^, CLA^+^ and CCR6^+^ and possess typical APC functions. In human skin explants, intradermal injection of Activin A increased the number of CD1a^+^ and Langerin^+^ cells in both the epidermis and dermis by promoting the differentiation of resident precursor cells. High levels of Activin A were present in the upper epidermal layers and in the dermis of Lichen Planus biopsies in association with a marked infiltration of CD1a^+^ and Langerin^+^ cells. This study reports that Activin A induces the differentiation of circulating CD14^+^ cells into LC. Since Activin A is abundantly produced during inflammatory conditions which are also characterized by increased numbers of LC, we propose that this cytokine represents a new pathway, alternative to TGFβ, responsible for LC differentiation during inflammatory/autoimmune conditions.

## Introduction

Langerhans cells (LC) are specialized dendritic cells (DC) normally found in the epidermis and mucosal stratified epithelia [1]–[5]. Contrary to myeloid and plasmacytoid DC, LC express the C-type lectin CD207 (Langerin), the major constituent of Birbeck granules, which represent the hallmark of LC [6]–[8]. LC also express a characteristic set of cell-surface molecules, such as cutaneous lymphocyte-associated antigen (CLA), E-cadherin and the CC chemokine receptor 6 (CCR6) [9]–[13]. As immature cells, their primary function is to sense the environment for danger signals and capture antigens; then LC undergo a process of functional and phenotypic maturation and migrate to the regional lymph nodes [5]; [10]; [14]; [15].

Several evidences suggest that LC are of myeloid origin and can differentiate from monocytes or CD34^+^ precursors [16]–[20]. While the generation of DC from monocytes requires GM-CSF and IL-4 only, the additional presence of TGFβ1 in the cytokine milieu appears to be essential for the development of LC [17]; [18]. Accordingly, TGFβ1-deficient mice display a severe defect in LC, but not in DC, development [21]. IL-15 is the only other cytokine known until now to skew monocyte differentiation toward LC-type DC; though, IL-15-derived LC are Langerin^+^ but lack Birbeck granules [22].

According to the current model of LC differentiation, in steady-state conditions LC are maintained locally by a stable renewable population present in the skin [23]–[25]. The existence of skin-resident LC precursors was postulated by Lareggina et al who described dermal CD14^+^ cells that express Langerin and CCR6 and are able to acquire LC features when cultured in the presence of TGFβ1 [26]. When the skin is exposed to inflammatory stimuli (UV rays, infections, allergens) LC increase their expression of class II MHC and costimulatory molecules and migrate to regional lymph nodes. In this situation of accelerated turnover, LC are replaced by blood-borne precursors such as inflammatory Gr-1^+^ monocytes recruited through a CCR2-dependent mechanism [23]; [27]; [28].

The epidermal environment can contribute to the attraction of precursors and to their differentiation into LC. Keratinocyte production of MIP-3α/CCL20 and TGF may direct CCR6^+^ LC precursors to the epidermis and induce their entry into the LC pathway, respectively [9]; [18]; [21]. However, LC differentiation could also depend on the dermal cytokine environment once LC precursors have entered the skin to colonize the dermis, or are trafficking through the dermis to the overlying epidermis. Indeed, emerging evidence support the concept that in certain skin pathological conditions a conspicuous expansion of the LC population occurs within the dermis [29]–[32] suggesting, that LC differentiation factors might also be produced within the dermal compartment.

Activin A is a member of the TGFβ1 family initially identified for its ability to control the secretion of follicle-stimulating hormone. Activin A is presently also known for its activity on growth and differentiation of various cell types during organogenesis, and for its role in wound healing, inflammation and tumor progression [33]–[36]. Activin A binds to specific transmembrane serine/threonine kinase receptors (ActRIB and ActRII) and to follistatin, a secreted protein that inhibits protein functions by sequestration [37]–[39]. Activin A is strongly induced after skin injury, probably by serum growth factors released upon haemorrhage and by macrophage-derived pro-inflammatory cytokines [40]; [41]. Transgenic mice overexpressing Activin A in the epidermis show strong hyperthickened epidermis, accelerated wound healing and enhanced scarring [42]; [43]. Conversely, in transgenic mice overexpressing the antagonist follistatin, skin wound closure is delayed and scar formation reduced [44]. Curiously, LC are strongly reduced in the skin of follistatin transgenic mice, suggesting a role of Activin A in LC biology [45].

This study shows that Activin A induces the differentiation of LC in vitro and ex-vivo and candidates Activin A as a new differentiation pathway that might be relevant in conditions characterized by the local production of Activin A and accumulation of LC.

## Results

### Activin A induces the differentiation of circulating monocytes into DC with phenotypic and ultrastructural features of LC

**Figure 1:**
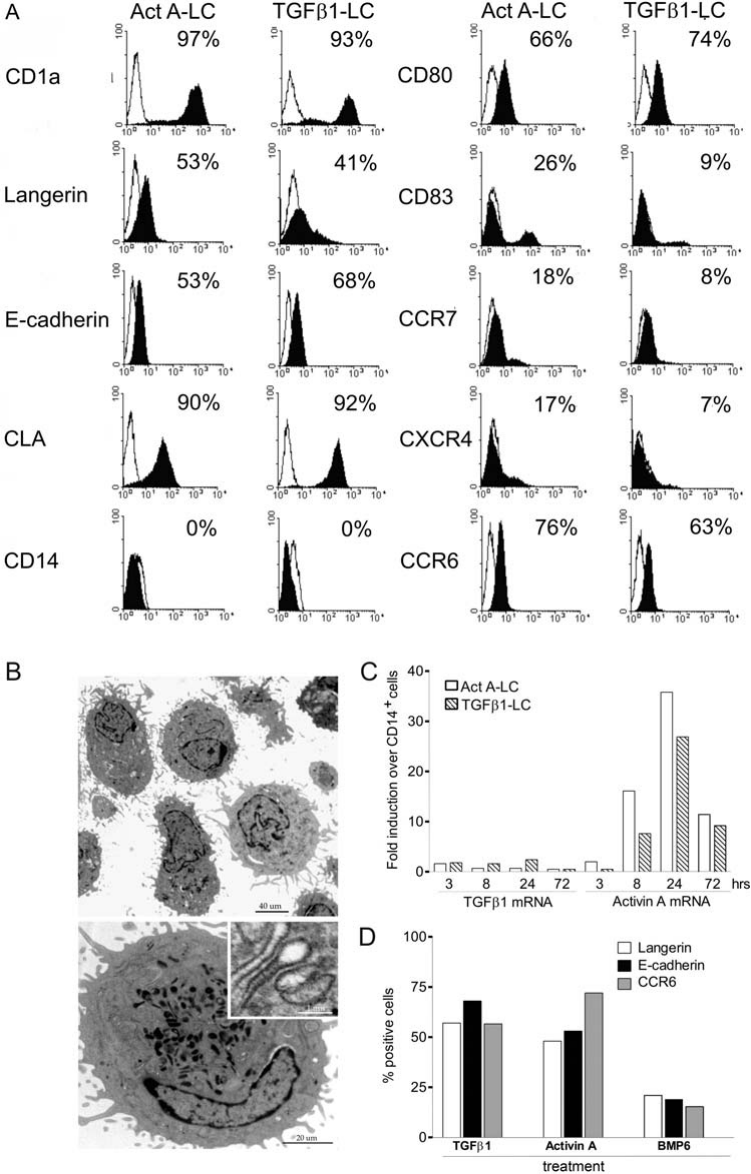
Activin A promotes Langerhans cell differentiation from human CD14^+^ monocytes. (A) Phenotypic analysis of monocytes cultured for 6 days with GM-CSF and IL-4 in the presence of Activin A (Act A-LC) or TGFβ1 (TGFβ1-LC). Cells were stained with the indicated moAbs (filled histograms) or isotype-matched negative control moAbs (open histograms). Percentages of positive cells are shown in the upper right corner of each histogram. The figure shows one experiment representative of at least five independent cultures. (B) Electron microscopy analysis of Act A-LC. Act A-LC exhibited abundant dendritic membrane protrusions and lobulated or indented nuclei (left panel, 3,000X, bar 40 µm). Cytoplasm presented a rough endoplasmic reticulum, many multilamellar organelles and numerous electron-dense structures reminiscent of Birbeck granules (right panel, 12,000X, bar 1 µm). The inset shows rod-shaped Birbeck granules (200,000X, bar 20 µm). (C) TGFβ1 and Activin A mRNA expression in Act A-LC and TGFβ1-LC cultures. Monocytes were cultured in the presence of Act A or TGFβ1 for the indicated time and the expression of TGFβ1 and Activin A mRNA was determined by real-time PCR, relative to GAPDH mRNA used as internal control. The expression level in freshly isolated monocytes was assumed as the 1.0 value. Similar results were obtained in three different donors. (D) Effects of different TGF family members on LC differentiation. Monocytes were cultured for 6 days with GM-CSF in the presence of 10 ng/ml TGFβ1, 100 ng/ml Activin A, or 100 ng/ml BMP6 and analyzed for Langerin, E-caderin and CCR6 expression by flow cytometry analysis. Data are representative of at least four independent cultures.

Highly purified monocytes were cultured for 6 days with Activin A in the presence of GM-CSF and IL-4, two cytokines that were shown to cooperate with TGFβ1 in the differentiation of monocytes to LC [16]; [18]. These cells (thereafter called Act A-LC to differentiate them from TGFβ1-LC) were CD14^−^ and expressed typical LC markers, such as CD1a, Langerin, E-caderin, CLA and CCR6. At day 6, Act A-LC presented an immature phenotype with a modest expression of CD80, CCR7 and CD83 (Fig. 1A). The presence of Birbeck granules, the hallmark of epidermal LC [7] was assessed by transmission electron microscopy on ultrathin-sections of Act A-LC cells (Fig. 1B). These cells displayed dendritic morphology with slightly off centred indented nuclei. The cytoplasm contained Birbeck granules, cytoplasmic organelles with rod like profile, electron-opaque central lamella and rounded ends. More open-ended tennis-racket shaped granules were also observed. Taken together this set of data indicates that Activin A, in the presence of GM-CSF and IL-4, induces the differentiation of circulating monocytes into LC. The possibility that the effect of Activin A could be due to the secondary induction of TGFβ, was investigated by real-time PCR. As shown in Figure 1C, TGFβ1 mRNA was barely detectable in Act A-LC cultures and similar results were obtained for TGFβ2 and TGFβ3 (data not shown); TGFβ1 was also weakly induced in TGFβ1-LC. On the contrary, Activin A mRNA was strongly upregulated by both Activin A and TGFβ1, suggesting the existence of an amplificatory loop. In agreement with mRNA levels, TGFβ1 concentration was below the detection limits (sensitivity 4.61 pg/ml) in Act A-LC supernatants, whereas Activin A was induced at 1.05 ng/10^6^ cells (n = 5, n = 6) in TGFβ1-LC. Furthermore, Act A-LC generation was not blocked by the addition of anti-TGFβ1 (data not shown), supporting a TGFβ1-independent differentiation of Act A-LC. Finally, it was tested whether bone morphogenetic protein (BMP-6), another TGF family member protein, could also induce LC differentiation. Figure 1D shows that BMP-6 did not sustain phenotypic LC differentiation, indicating that the ability to induce LC differentiation is not a general feature shared by all TGFβ family member proteins.

### Phenotypical and functional characterization of Act A-LC

**Figure 2:**
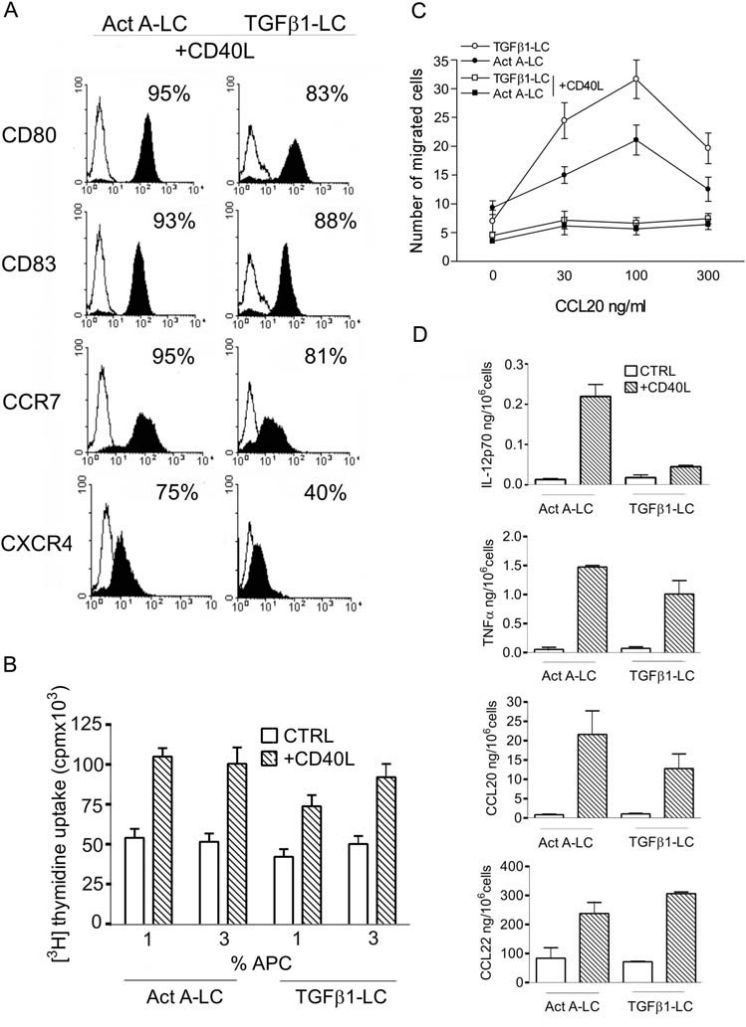
Phenotypical and functional characterization of CD40L-activated Act A-LC (A) Expression of maturation markers by Act A-LC. Act A-LC were incubated with CD40L-transfected fibroblasts for 40 hrs and stained with anti-CD80, CD83, CCR7 and CXCR4 moAbs (filled histograms) or isotype-matched negative control Abs (open histograms). Results obtained with TGFβ1-LC are also shown for comparison. The percentage of positive cells is reported in each panel. Data shown are representative of three independent experiments. (B) Allostimulatory capacity of Act A-LC. Irradiated immature or CD40L-matured Act A-LC (or TGFβ1-LC) were cultured with 2×10^5^ allogeneic purified T cells. Proliferation was assayed as uptake of [H^3^]thymidine added in the last 16 hrs of a 6-day culture assay. Results are expressed as mean counts per minute (cpm)±SD of one representative experiment performed in triplicate. Values are at the net of T cell proliferation in the absence of DC (3250±250 cpm). (C) Act A-LC migrate in response to CCL20. Immature or CD40L-mature Act A-LC or TGFβ1-LC were applied to the upper wells of the chemotaxis chamber. CCL20 was added to the lower level of the chamber. The number of cells migrated to the lower chamber was counted. Each assay was performed in triplicate and the results are expressed as the mean±SD number of migrated cells (representative of three experiments). (D) Cytokine release by Act A-LC. Immature or CD40L-mature Act A-LC or TGFβ1-LC were assessed for their ability to release the indicated cytokines by ELISA. Results are the average determination (±SD) of four independent experiments.

Membrane phenotype and functions were tested in immature and CD40L-mature Act A-LC. As shown in Figure 2A CD40L-activated Act A-LC showed increased surface expression of CD80 and CD83 as well as the expression of CXCR4 and CCR7, two maturation-associated chemokine receptors; these data are consistent with the acquisition of a mature phenotype. Immature Act A-LC efficiently stimulated T cell proliferation and their allostimulatory capacity was further enhanced upon maturation (Fig. 2B). In agreement with the expression of CCR6 (Fig. 1A and data not shown), immature, but not mature Act A-LC migrated in response to CCL20 (Fig. 2C). On the contrary, CD40L-mature cells migrated in response to CCL19, one of the CCR7 ligands (data not shown). Finally, CD40L-activated Act A-DC secreted IL-12p70, TNF, CCL22 and CCL20 in a similar manner to TGFβ1-LC, with the exception of IL-12 which was consistently produced at higher levels by Act A-LC (Fig. 2D). Therefore, LC generated in the presence of Activin A have the capacity to undergo a full maturation process based on membrane phenotype, migration and functional properties.

### Activin A induces the generation of Langerin^+^ cells ex-vivo in human skin explants

**Figure 3:**
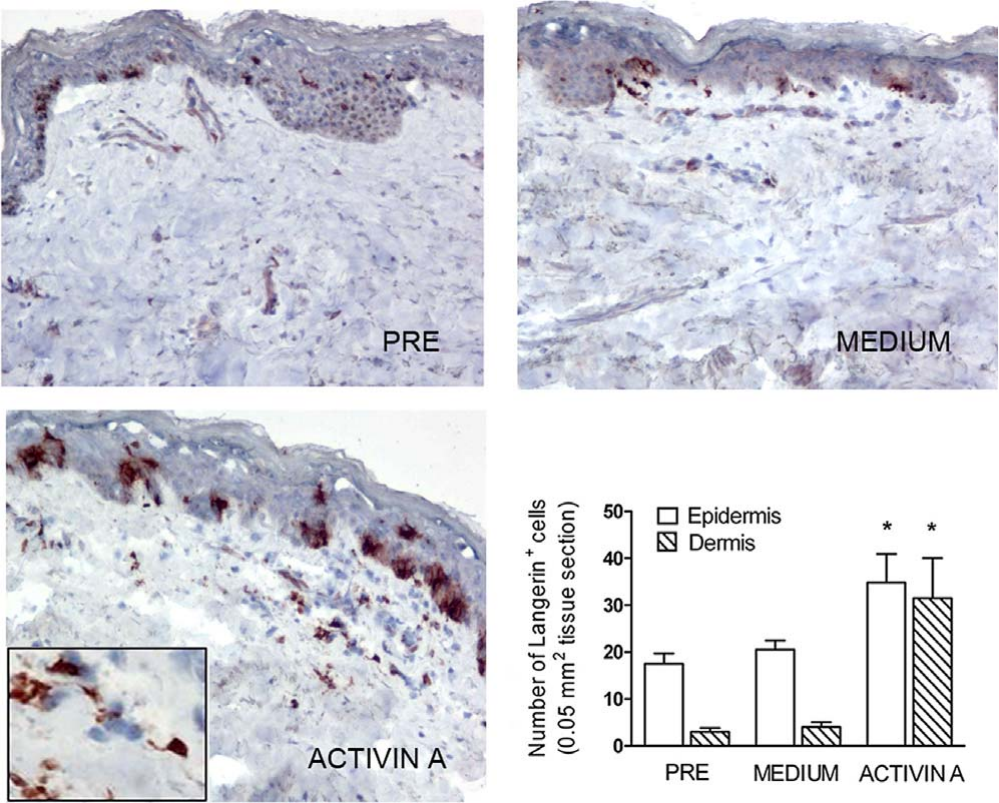
Intradermal injection of Activin A induces the differentiation of dermal and epidermal Langerhans cells in human skin explants. Langerin expression was evaluated in the epidermis and dermis (full thickness skin explants) of skin explants, untreated and 72 hrs after i.d. injection of medium or 100 ng Activin A (magnification 100X, inset 400X) The number of Langerin^+^ cells were quantified in skin explants by evaluating six different skin sections (0.05 mm^2^/field; means±SD). * p<0.05 by Student's t test vs. medium (lower right panel).

To investigate the ability of Activin A to induce LC differentiation within the skin milieu, skin explants were intradermally injected with Activin A and subsequently cultured in six-well culture plates at the air-medium interface with the epidermis side up [46]. Explants were subsequently removed and examined by immunohistochemistry. As expected, fresh, untreated skin explants revealed several Langerin^+^ cells within the epidermis (Fig. 3) and a similar picture was observed following the injection of medium. Instead, the inoculation of 100 ng Activin A led to a profound increase in the number of Langerin^+^ cells both in the epidermis and in the dermal layer, with a maximal induction observed 72 hrs after the injection. The increase of the number of Langerin^+^ cells was of about 2-fold and 10-fold (n = 6) in the epidermis and dermis, respectively, and was statistically significant with respect to control skin (p<0.05, by Student's t-test; Fig. 3). The number of CD1a^+^ cells also increased in parallel to Langerin expression (data not shown). These results show that Activin A is able to induce the differentiation of LC precursors resident within normal skin.

### Activin A promotes LC differentiation from precursors cells present in the dermal layer

**Figure 4:**
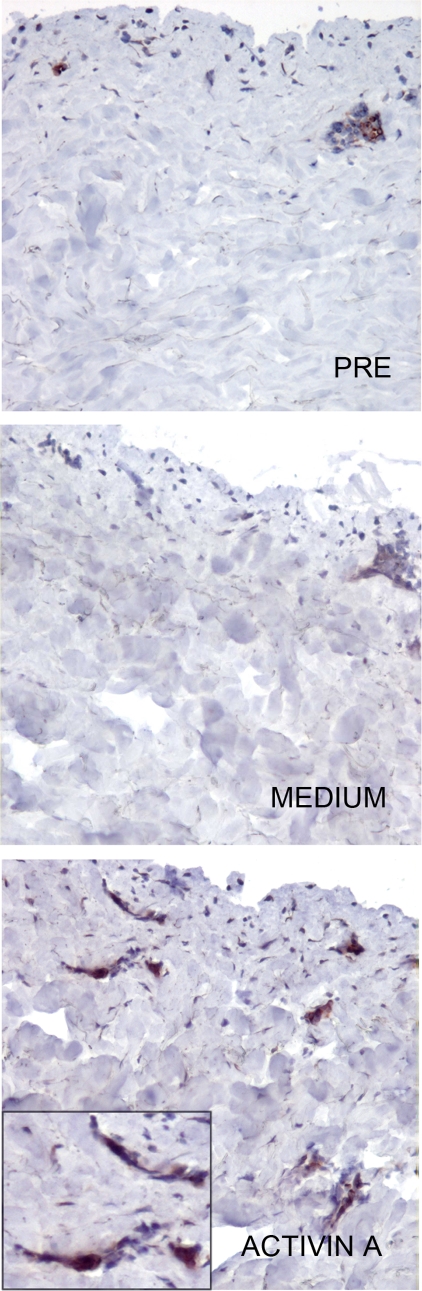
Activin A induces Langerhans cells differentiation in epidermis-depleted skin explants. Langerin expression was evaluated in the dermal layer, separated from skin explants by dispase digestion and subsequently treated for 72 hrs after i.d. injection with 100 ng Activin A (magnification 100X, inset 400X.).

To exclude that the increased number of LC observed in the dermis following Activin A injection could be due to migration of LC from the epidermis, the dermis was separated by dispase digestion and thereafter injected with Activin A. Also under these experimental conditions, Activin A inoculation strongly increased the number of Langerin^+^ cells (Fig. 4). To better address the potential of dermal precursors to differentiate into LC under the influence of Activin A, skin migratory cells were recovered from dermal layers and subsequently cultured in the presence of Activin A. At day 6 of culture, a consistent number of cells, 15% and 20% were positive for CD1a and Langerin, respectively. Conversely, no Langerin^+^ cells could be detected in the absence of Activin A. Altogether, these data show that skin precursors, present within the dermis, can be induced to differentiate into LC by Activin A.

### Dermal accumulation of Langerhans cells in lichen planus is associated with abundant production of Activin A

**Figure 5:**
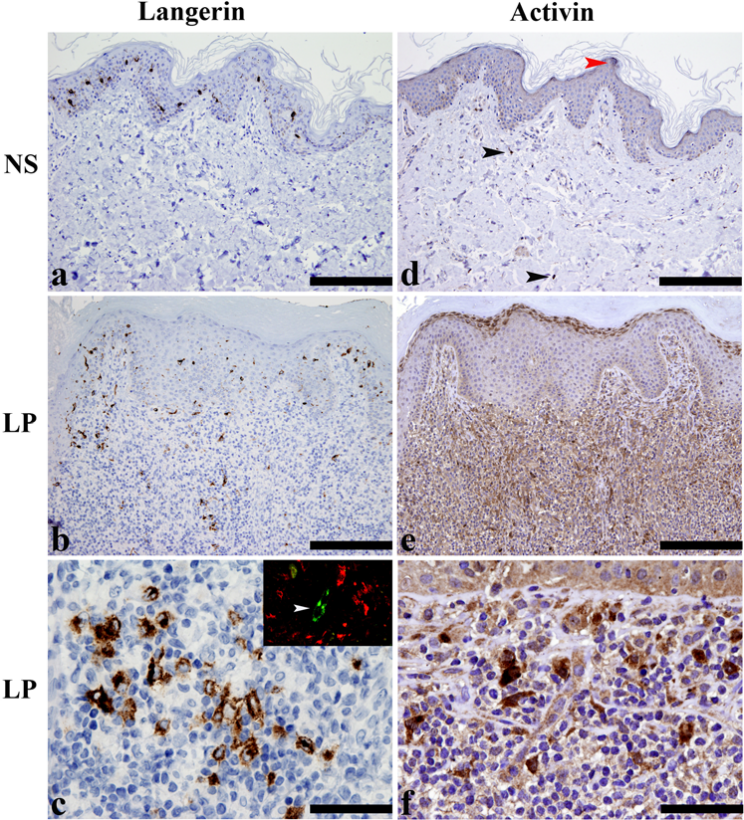
Dermal accumulation of Langerhans cells in lichen planus is associated to abundant production of Activin A. Sections from normal skin (NS) (*a* and *d*) and lichen planus (LP) (*b, c, e, f*) biopsies were stained for Langerin (*a*–*c*) and Activin A (*d–f*). In normal skin, Langerin^+^ cells are regularly distributed in basal and suprabasal layers and show multiple fine dendrites; no positive cells are detectable in the dermis (panel *a*). In LP biopsies, in addition to intraepidermal LC, accumulation of Langerin^+^ cells is observed in the dermis within the dense monuclear cell infiltrate (panel *b*). At high power view, Langerin^+^ cells show an ovoidal/dendritic shape (panel *c*) and are found surrounding Factor VIII^+^ dermal blood vessels (arrow head, inset in *c*). Serial sections from the same tissue blocks were stained for Activin A. Normal skin (panel *d*) showed weak intraepithelial reactivity (red arrow head); in the dermis, mast cells and occasional spindle cells were positive for Activin A (black arrow heads). In LP, Activin A was strongly induced in the superficial layers of epidermis; in the dermis, a diffuse reactivity can be observed in numerous cells within the inflammatory infiltrate (panel *e*). This cell population includes endothelial cells and a mixture of non-lymphoid mononuclear cells (panel *f*). Magnification 100x (*a, b, d, e*; scale bar 200 micron) and 400x (*c, f*; scale bar 50 micron).

Although LC are predominantly confined to the epidermis, we and others have recently documented that in certain pathological conditions, such as lichen planus, LC are also abundantly found in the stromal compartment [29]; [30]; [32]. As shown in Fig. 5 (panel a), in normal skin and mucosa LC are regularly distributed within the epithelium in the basal and suprabasal layers and are easily recognized based on their dendritic morphology and expression of Langerin [7]. On the contrary, in the large majority of the lichen planus cases investigated (32/34) variable numbers of Langerin^+^ dendritic cells were identifiable in the stromal compartment (Fig. 5, panel b), distributed as sparse cells or clusters within the mononuclear infiltrate (Fig. 5, panel c). Of interest, many of these Langerin^+^ cells were detected in close proximity to blood vessels, as shown by double immunofluorescence for Langerin and Factor VIII-related antigen (Fig. 5, panel c, inset). In normal skin a weak reactivity for Activin A was detected in epidermal keratinocytes and rare spindle cells (likely representing dermal macrophages or dendritic cells); a stronger positivity was also observed in scattered mast cell [47] (Fig. 5, panel d). Compared to normal skin, lesional skin and mucosa from lichen planus biopsies showed strong induction of Activin A in the upper layers of the epidermis and, particularly, in the stromal compartment (Figure 5, panel panel e). In the latter, Activin A was mostly produced by non-lymphoid mononuclear cells, endothelial cells and mast cells (Figure 5, panel f)

## Discussion

This study reports that Activin A, a protein abundantly produced in the skin during normal and pathological wound healing [40]; [47] and inflammatory/autoimmune diseases (this study), induces the differentiation of human CD14^+^ monocytes in Langerin^+^, Birbeck granules^+^, E-cadherin^+^, CLA^+^ and CCR6^+^ cells. LC originate in vitro from CD34^+^ bone marrow precursors [16]; [17]; [19]; [20]. Monocytes also represent LC blood precursor cells in vitro and in vivo [18]; [22]; [27]; [28]. Indeed, CD14^+^ cells can be induced to differentiate into LC by a cytokine combination including GM-CSF, IL-4 and TGFβ1 [18]. The crucial role of TGFβ in LC differentiation has been clearly documented by the observation that TGFβ1 null mice are devoid of LC [21]. More recently, it was shown that the cytokine milieu present at the inflammatory site may favour LC differentiation through an alternative pathway [22]; [28]. Indeed, in vitro experiments have shown that IL-15, in cooperation with GM-CSF, induces the differentiation of monocytes into cells that express LC markers, such as E-cadherin, CCR6 and Langerin but lacking the expression of conventional Birbeck granules [22]. The present study adds Activin A to the limited list of cytokines that possess the potential to promote LC differentiation. Activin A is a member of the TGFβ family and shares some of the intra-cellular signalling pathways with this cytokine [48]. However, the ability to induce LC differentiation is not a general feature shared by all TGFβ family member proteins, as documented by the lack of activity of BMP-6 in our assay conditions. The selective action of Activin A versus BMP-6 is likely to be due to the usage of specific transmembrane receptors and the activation of different signalling pathways [49]; [50].

LC form a cellular network in the epidermis that constitutes the first immunological barrier against pathogens and dangerous insults. Following antigen capture, LC leave the epidermis by a mechanism that depends on the expression of chemotactic receptors, adhesion molecules and proteases [10]; [14]; [51]. Emigrating skin cells enter lymphatic vessels located in the superficial dermis to finally reach draining lymph nodes where they present processed antigens to naïve T cells [52]. During this migratory process, LC acquire a mature phenotype that is associated with the expression of homing receptors, co-stimulatory molecules and the ability to release several cytokines [10]; [52]; [53]. The results presented in this study show that LC generated in the presence of Activin A are fully competent to undergo a maturation process, as evaluated by the expression of CCR7 and the downregulation of CCR6, the expression of CD80 and CD83, the ability to induce T cell proliferation and to secrete high levels of chemokines (i.e. CCL20 and CCL22) and cytokines (TNF-α, IL-12p70).

LC are normally confined to the basal and suprabasal layer of the epidermis and stratified epithelia of mucosal surfaces. These cells are clearly distinct from dermal/interstitial DC which lack Birbeck granules and Langerin expression, but express DC-SIGN, Factor XIIIa and more rarely CD1a [54]–[58]. The current view proposes that under steady-state conditions, dermal-resident CD14^+^ precursor cells have the potential to migrate to the epidermis in response to CCL20 and there, in the presence of keratinocyte-derived TGFβ, differentiate into immature resident LC characterized by a weak T cell stimulatory activity. In the presence of the cytokine rich milieu that characterizes many pathological conditions, migratory CD14^+^ cells further differentiate into more mature LC which possess a higher antigen presenting activity [25]; [26]; [28]. In order to evaluate the potential of skin resident precursor cells to differentiate into LC in response to Activin A, we performed experiments in which skin biopsies were inoculated ex-vivo with Activin A and further incubated in vitro [46]. The immunohistochemical evaluation of these skin explants clearly show that the injection of Activin A induced a strong increase in the number of Langerin^+^/CD1a^+^ cells in both the epidermal and dermal compartments in a time-dependent manner. Due to the experimental conditions employed, Langerin^+^ cells must have originated from skin-resident precursor cells. Further we show that Langerin^+^ cells can be induced to differentiate by Activin A within the dermis in the absence of epidermis. In agreement with these results, cells emigrated from skin explants could also be induced to differentiate into Langerin^+^/CD1a^+^ cells by the presence of Activin A in vitro. These findings are compatible with the description of CD14^+^, Langerin^+^ LC precursors located in the superficial and deep dermis, predominantly in perivascular areas [24]; [26]. Although the precise characterization of Activin A-responsive dermal LC precursors is beyond the aim of the present study, these results clearly document that Activin A can induce the local differentiation of dermal LC precursors. In this contest it is interesting to note that Stoitzner et al. reported that in mice overexpressing in the follistatin, the natural Activin A antagonist, the number of LC is reduced [45].

Although Activin A is very weakly expressed in normal skin, its expression was dramatically increased in lichen planus biopsies. In this condition, Activin A was expressed both in the superficial epidermis and in the dermis by stromal cells, infiltrating leukocytes, including mast cells and some blood vessels. As expected on the basis of previous work [29]–[32], lichen planus biopsies show a prominent increase in the number of LC which were present in the deep and superficial derma and in the epidermis. Of note, LC were often present in clusters localized around Factor VIII^+^ blood vessels, suggesting the involvement of newly vascular-recruited precursor cells. Although these data generated in a human disease do not allow formal conclusions, it is tempting to speculate that during certain pathological conditions characterized by the local expression of Activin A and inflammatory cytokines (such as GM-CSF and IL-4), dermal LC precursors, or newly recruited blood elements, can be induced to differentiate to LC within the stromal compartment. This model may help to explain the origin of LC localized in the deep dermal layers, away from the epidermis and from superficial lymphatic vessels.

Lichen planus is an autoimmune disease characterized by a prominent cellular infiltrate mainly composed of DC, LC, cytotoxic T lymphocytes and NK cells, localized in close proximity of apoptotic keratinocytes [29]–[32]. The involvement of the TGFβ family members in this pathology is suggested by several observations. First, BMP-4 is upregulated in the epithelium of lichen planus [59]. Second, several lines of evidence suggest that the TGFβ activation and/or signal transduction pathway might be defective in this disease. Indeed, lichen planus is associated with epithelial hyperproliferation, a situation that is usually negatively controlled by TGFβ, and this is consistent with the identification of TGFβ positive T cells in the sub-epithelial lymphocytic infiltrate but not within the epithelium itself. It is therefore of interest to note that this defective TGFβ pathway is associated with a high expression of Activin A (this study). In this context it is likely that Activin A may have a prominent role in LC differentiation in lichen planus. Furthermore, Activin A may contribute to the pathogenesis of lichen planus by favouring epithelial hyperplasia.

In summary, this study presents a new model in which Activin A induces the differentiation of circulating CD14^+^ cells into LC. Since Activin A is abundantly produced during certain inflammatory conditions, we propose that this cytokine represents a new pathway, alternative to TGFβ, responsible for LC differentiation during inflammatory/autoimmune conditions.

## Materials and Methods

The study was conducted in accordance with a protocol approved by the Spedali Civili of Brescia Institutional Ethical Board (Brescia, Italy) and the Board of the CTO Hospital (Turin, Italy); written informed consent was obtained from all patients.

### Cell cultures

CD14^+^ monocytes were isolated from buffy coats (Centro Trasfusionale Brescia, Italy) by positive magnetic separation using CD14 immunomagnetic beads (Miltenyi Biotec, Auburn, CA) [60]. To generate LC, monocytes were cultured for 6 days in 6 wells tissue culture plates (Costar, Corning, Cambridge, MA) in 10% heat-inactivated FCS RPMI 1640 (Sigma, St. Louis, MO) supplemented with 100 U/mL penicillin, 100 µg/mL streptomycin, 2 mM L-glutamine. GM-CSF 100ng/ml and IL-4 10 ng/ml were added at day 0. 10ng/ml TGFβ1 or 100 ng/ml Activin A were also added at day 0. All cytokines were from Peprotech, Rocky Hill, NJ. Half the culture medium was replaced with fresh medium containing cytokines on day 2 and 4. LC maturation was induced by incubation with CD40L-transfected J558 cells (1∶4 ratio) for 24 hrs.

### Flow cytometric analysis

Surface phenotype analysis was performed using the following antibodies: phycoerythrin (PE)-conjugated anti-CD1a (anti-CD1a-PE), anti-Langerin (CD207)-PE, anti-E-cadherin, and anti-CD83-PE (Immunotech, Marseille, France); anti-human leukocyte antigen (HLA)-ABC-PE and FITC-conjugated anti-CD1a (anti-CD1a-FITC; Dako, Glostrup, Denmark); anti-HLA-DR-PE, anti-CD80-PE, (BD PharMingen, San Diego, CA); anti-CD86-PE, anti-CD14-PE, anti-CC chemokine receptor 7 (CCR7)-PE, and anti-CCR6-FITC (R&D Systems). Anti-CLA-FITC rat mAb (BD PharMingen) was also used. Mouse immunoglobulin G1 (IgG1)-PE, mouse IgG1-FITC, rat anti-mouse IgG1-FITC, and rat IgG2a-PE were from BD Pharmingen. Purified mouse IgG1 (R&D Systems), rat IgM-FITC, mouse IgG2b-PE, mouse IgG2a-PE (BD PharMingen), or mouse IgG2b-FITC (Beckman Coulter, Hialeah, FL) were used as an isotype control. Cells were analyzed with a FACScan flow cytometer (Becton Dickinson, San Jose, CA) using CellQuest software.

### Transmission electron microscopy

10^6^ cells were fixed with 2.5% glutaraldehyde in 0.1 M cacodylate buffer (pH 7.4) and postfixed with 1% osmium tetroxide in 0.1 M cacodylate buffer (pH 7.4). Next, cells were dehydrated through a graded series of ethanol and embedded in araldite (Poersch, Frankfurt, Germany). Ultrathin sections were counterstained with uranyl acetate and lead citrate and were examined with a Zeiss electron microscope (EM 906, Zeiss, Oberkochen, Germany).

### Evaluation of DC functions

Irradiated immature and CD40L-stimulated LC were added in triplicate in graded doses to 2×10^5^ purified allogeneic T cells in 96-well round-bottom plates. [^3^H]Thymidine incorporation was measured on day 5 after a 16-h pulse (5 Ci/µmol; Amersham Biosciences, Freiburg, Germany). Human IL-12p70, Activin A, TGFβ1, MDC/CCL22, TARC/CCL17, MIP-3α/CCL20 protein levels in the culture supernatants were measured by sandwich ELISA (R&D Systems, Minneapolis, MN). DC migration was evaluated using a 48-well microchemotaxis chamber (Neuroprobe, Pleasanton, CA) with 5-µm pore size polyvinylpyrrolidone polycarbonate filters (Neuroprobe) as previously described [61].

### RNA purification and real time RT-PCR analysis

RNA samples, extracted by using TRIzol (Invitrogen, Carlsbad, CA), were treated with DNase (Invitrogen) and single-stranded complementary DNA (cDNA) was synthesized by reverse transcription of 2 µg total RNA using random hexamers and the Superscript First-Strand Synthesis System for RT-PCR (Invitrogen). The cDNAs were then amplified in duplicate by real-time PCR using the Platinum SYBR Green (Invitrogen) in a final volume of 25 µl and quantitative analysis was carried out as previously described [47]. The sequences of primers were as follows: Activin A (sense 5′-GCA GAA ATG AAT GAA CTT ATG GA-3′; antisense 5′-GTC TTC CTG GCT GTT CCT GAC T-3′), TGFβ1 (sense 5′-GCG TGC TAA TGG TGG AAA-3′; antisense 5′-CGG TGA CAT CAA AGA TAA CCA C-3′), β-actin (sense, 5′-GTT GCT ATC CAG GCT GTG-3′; antisense, 5′-TGT CCA CGT CAC ACT TCA-3′).

### Skin explant cultures

Skin specimens were obtained from patients undergoing corrective breast or abdominal plastic surgery. Cytokines were injected into the dermis with a MicroFine insulin syringe (29 gauge needle) in the indicated amounts and in a total volume of 20 µl. At the site of injection, a ∼5-mm wheal appeared and a 6 mm punch biopsy was taken. For immunohistochemistry, skin biopsies were cultured at air-medium interface with the epidermis side up in a six-well culture plate (Costar) on sterilized stainless steel grids covered with a filter (Millipore, Bedford, MA; 45 µm), at 37° C in 5% CO_2_ humidified air [46]; [62]. At the indicated times, the explants were harvested, snap frozen, and stored in liquid nitrogen. To study phenotypic development of epidermal and dermal DC separately, the epidermal and dermal layers were separated by dispase digestion for 1 h at 37°C (Dispase grade II, 50mg/ml; Roche). To obtain emigrated skin cells, the epidermal and dermal layers were placed directly in 1 ml culture medium (floating with epidermis side up) in a 48-well culture plate (Costar) with 100 ng/ml Activin A. After 7 h, migratory cells were collected, counted in hemocytometers using trypan blue exclusion and cultured in the presence of GM-CSF and Activin A for 6 days.

### Tissue specimens and staining procedures

Paraffin embedded tissue blocks were taken from the archive of the Department of Pathology of the University of Brescia/Spedali Civili di Brescia; tissues included normal skin, oral (13 cases) and cutaneous (21 cases) lichen planus and granulosa cell tumors (3 cases). Four micron tissue sections were used for immunohistochemical staining using primary Abs to the following antigens: human Activin A (clone E4, dilution 1∶50, Serotec, Oxford, UK), human Langerin (clone 12D6, 1∶200, Vector Laboratories, Burlingame, CA), human Factor VIII-related antigen (rabbit polyclonal, 1∶100, Neomarkers, Westnghouse, CA). Upon antigen retrieval with microwave treatment (3 cycles of 5 min×750 W) or thermostatic bath (40′ at 89°) in EDTA buffer solution, reactivity was revealed using Real EnVision Mouse/Rabbit-HRP (DakoCytomation, Glostrup, Denmark) or SuperSensitive IHC Detection System (BioGenex, San Ramon, CA). For double-immunofluorescence staining anti-human Langerin and Factor VIII-related antigen were revealed respectively using a goat anti-mouse IgG2b (1∶75, Southern Biotek, Birmingham, AL) followed by Streptavidin Texas Red (1∶75, Southern Biotek) and a FITC-conjugated swine anti-rabbit (1∶30, Dako Cytomation). For immunohistochemical staining of Activin A, positive tissue control was represented by granulosa cell tumors that strongly expressed this protein [63]; no reactivity was observed when omission of primary Ab and irrelevant isotype matched primary antibody were used. Immunostained sections were independently examined by two pathologists (WV and FF); digital images taken using the Olympus BX60 microscope and a DP-70 Olympus digital camera were processed using Analysis Image Processing software.
